# Review of Books

**Published:** 1924-03

**Authors:** 


### REVIEWS OF BOOKS.

### AN ARMY SURGEON OF THE SEVENTEENTH CENTURY.

**Master Johann Dietz. The Faithful Description of His Life.** Written by himself. Translated from the German by Bernard Miall. Illustrated. (George Allen and Unwin. 12s. 6d. net.)

In the year 1665 when Samuel Pepys was writing for his own private delectation the diary which has given him a place among the Immortals, there was born in the City of Halle a worthy citizen who, at the end of a long and adventurous life, set down his story for the edification of his friends. Johann Dietz, the son of a prosperous rope-maker and corn-chandler, was apprenticed in 1681 to a barber-surgeon, and the picture which he gives of the life of an apprentice in Germany is probably a faithful representation of similar conditions in England. The menial duties assigned to him, manuring the garden, splitting the wood, lighting the fires, drawing the water, and the blows and heavy-handed correction appear to have loomed large in his memory in after years.

In the second year of his apprenticeship bubonic plague, of which he gives a graphic account, made its appearance in Halle. Fifty or sixty persons died every day in the city, round which was drawn a cordon of soldiers, an attempt to pass through it being punishable by death. When the young surgeon-apprentice finally succumbed he was taken by his father into a barn, locked in and left to his fate. Fortunately he survived, and at the conclusion of his apprenticeship he set off to try his fortune in Berlin. The real interest of his story begins with his appointment as an army surgeon attached to the artillery for the Hungarian campaign against the Turks, 1684 to 1686. A weary and dangerous march it must have been. Mustering 12,000 strong at Berlin, the expeditionary force marched by Frankfurt, Breslau and over the Alps by the Jablunka Pass into the Trentino, and thence by Kremmitz over the Danube at Grau to Neuhausel, then recently recaptured from the Turks, to the beleagerment of Buda Pesth. Dietz's account of his experience of trench warfare has an extraordinarily modern ring about it:—

> I sat there in a deeply-dug hole, protected from the bombs by turves and rafters to one side of the door, for bullets came in at the door every moment passing me and plunking into the wall. Bombs, too, fell on our bastion, but rolled down before they burst. . . . The bullets were raining round me so that I did not know where to turn, . . . . but greater still were the shock and horror when I entered the communication trenches. There the bullets were flying and whistling about me, and then I saw how one man after another fell shreiking. Then it was "Surgeon! Surgeon!" from this man and that.

War is a savage and bloody business still, but the cruelty and insensate bloodlust which then wreacked itself upon the defeated foe, women and children alike was horrible beyond description. With the fall of the fortress the campaign came to an end, and at length a pitiful remnant of the army, reduced to less than 3,000 men, reached the gates of Berlin, still carrying typhus with them. "The Hungarian sickness hung round our necks like a curse." Unconsciously Dietz gives a clue to the cause :—

> And there was not a day but numbers of our soldiers died, so that our position was a pitiable one, for we were getting no pay. My own coats and shirts were in tatters. And although I had to tend the sick and make them sound again, I was so full of vermin that I got no rest of a night. On this account I had to creep stark naked into the hay at night, for only then could I get any rest.

His next chance of service came when the King of Denmark sent 8,000 men to England to serve against France. Unfortunately, however, Dietz exchanged with a Danish surgeon and thus lost for us the opportunity of an interesting record of his impressions of England. Instead he accompanied a military force to Copenhagen. Returning to Hamburg, his adventurous spirit led him to throw in his lot with the crew of a Dutch whaler, and within a week he was sailing in the *Hope of Rotterdam* on the way to Spitzbergen and the Arctic Ocean. He seems to have made several voyages, for he mentions visits to Iceland, Novaya Zembla, Lapland and Bergen, and he gives us a very vivid account of harpooning whales, reindeer-stalking, bear-hunting and an encounter with a French privateer, whose salvo of guns brought down their mizen mast.

But of adventures as of other pleasures there cometh satiety at last, and Dietz made up his mind to marry and settle down. Much of the second half of his reminiscences is concerned with his matrimonial troubles, his quarrels with the various vested interests of barber-surgeons' guilds with which he came into conflict, and with numerous law-suits. He lived to become Guildmaster of the Barber-Surgeons of Halle and churchwarden of St. Moritz, the church in which he had been baptised. He died in 1738 within a few feet of the spot where he had first seen the light seventy-two years before. The book is a fascinating description of middle-class life and of adventure. It has, moreover, the additional merit of being profusely illustrated by numerous reproductions of contemporary prints. It is well documented and supplied with useful explanatory notes. The translation is excellent.

### IS THERE AN UNCONSCIOUS MIND ?

**Psycho-Analysts Analysed.** By P. McBride, M.D., F.R.C.P.E., F.R.S.E. (Heinemann. 3s. 6d. net.)

Dr. McBride has little belief in the more extreme claims of the psycho-analysts, and none whatever in their postulate of the existence of the "Unconscious Mind," and he not only says so with great plainness but brings Sir Bryan Donkin into court to back him up. Both of them make excellent swordplay with the abominable jargon of the new school—their "complexes" and "unconscious repressions," their "will to believe," their "Psyche," and the rest of what we may be forgiven for calling their stock-in-trade. Let us not be misunderstood. That there is such a thing as psychology, and that it can be analysed, is not in question. What is in question is whether, on balance, psycho-analysis does more harm than good, and whether many of those who practise this pseudo- "Science" know much more about it than the confiding persons whom they treat. That it often does harm is certain ; how often it does good is still in doubt. The foundation-stone of it all is the Unconscious Mind. If that does not, as a fact, exist the entire psycho-analytic edifice falls to the ground. That it does exist neither has been nor can be proved ; yet the patient must believe in it and the practitioner must believe in it. Can we then be surprised that great numbers of the well-learned and the scientific like not this empirical security ?

In the whole of the Freudian conception there is nothing more disagreeable, nothing, indeed, more unbelievable, than the insistence upon all the all-pervadingness of sex. According to Freud, even babies are " polymorph sexual perverts," a notion as absurd in itself as its terminology is grotesque. A little girl who says, " I want to sit on daddy's knee " is suffering from a sex-complex. Nothing could be more wicked than an apparently innocent dream ; in fact, the more " conspicuously innocent " a dream, the more certainly does it " embody coarse erotic wishes." The language is that of Freud himself, and the unsophisticated reader may well ask himself how Freud knows ? Either he has had a vision from heaven—or elsewhere—or he is no better a spiritual guide than Joanna Southcott or Brothers the Prophet. Between people who believe such things and those who believe that fairies can be photographed there is nothing whatever to choose. To dream of the number three is really to dream of something sexual ; if you go upstairs in a dream you are after no good ; while to dream of opening a door and entering a room means that your Unconscious Mind is, for the time being, in a highly sexualised condition. We cannot doubt that Bishops, and even Popes, are capable of dreaming such things, and it is quite uncomfortable to realise what very bad men they must be, without knowing it. It would be infinitely more natural to conclude that dreaming of three means that you are going to receive a cheque for three pounds, or merely a postal order for three shillings, or that opening the door of a room implies no more than that you are making an afternoon call or " going out to tea." But these innocencies will not do for the Freudans and the Jungites. Freud tells us that he has reached his conclusions by observation ; but it is impossible to observe the invisible, and, in any case, he might just as easily have observed it all quite differently. But then psycho-analysis would have been not nearly so interesting. As it is the field is wide open to the Freudians to treat our nocturnal fantasies as matters of the gravest moment instead of, as so often happens, their being the result of too much dinner or the wrong kind of dish.

Dr. McBride presses the psycho-analysts very hard —perhaps, sometimes a trifle too hard. He smites them hip and thigh, using their own language as weapon ; but they are not all extravagant, or merely inventive, and the saner aspects of their theories are worthy of careful scientific treatment. We are not suggesting that Dr. McBridge fails to treat them scientifically, but his condemnations are apt to be a trifle sweeping, and we shall look with animated expectancy to seeing him put through the Freudian mincing-machine.

### BLOOD PRESSURE.

**High Blood Pressure : Its Variations and Control.** A Manual for Practitioners. By J. F. Halls Dally, M.A., M.D., B.C. Cantab., M.R.C.P. (Heinemann. 10s. 6d.)

The author's choice of the title, *High Blood Pressure*, is rather unfortunate. His comprehensive discussion includes low as well as high blood pressure, and the former may, indeed, come to be regarded as being as serious as the latter. Dr. Dally's aim has been to present to the general practitioner a summary of modern views on blood pressure, and his bibliography shows that he has gathered his own knowledge from a wide field. The book is a sign of the times. A generation ago the general practitioner did not dream of using the sphygmomanometer in his routine work any more than his grandfather dreamed of using a clinical thermometer. The time is probably not far distant when the sphygmomanometer will be considered as indispensable to the outfit of a practitioner as the clinical thermometer, and the doctor who relies on tactile impressions from feeling the pulse will be considered as much out of date as his colleague who takes a patient's temperature by patting his skin. Once the technique of measuring the blood pressure has been mastered, it takes less time and trouble than testing the urine.

But the sphygmomanometer is worse than useless unless two conditions are fulfilled. The practitioner must learn how to take accurate readings, and he must know what these readings indicate. On these two essential matters the author is most helpful, and even when he digresses from his main theme he is instructive as well as entertaining, and in his advice with regard to exercise and the choice thereof he quotes Lord Palmerston's famous aphorism : " The best thing for the inside of a man is the outside of a horse." Separate chapters are allotted to the relation of blood pressure to pulmonary tuberculosis and life assurance. Dr. Dally's book is particularly to be recommended to the practitioner who has not had time or opportunity to digest the abundant literature of this subject during the last decade, and who would like in a few hours to master the principles and practice of blood pressure measurement.

### MALADY AND MELODY.

**Music, Health and Character.** By Agnes Savill, M.D. (John Lane. 7s. 6d. net.)

As an autobiography of musical experience alone this book is distinctly out of the common, but it is more than that. The first portion is in reality a profound psychological study in which the reader is admitted into the secret of the author's conversion from a mood of downright antipathy to that of a love for all that is appealing and beautiful in musical art. As instruments in effecting this change Dr. Savill cites the player-piano and the less pretentious but more universal gramophone. We are not primarily concerned here with musical criticism, but it is impossible to refrain from commenting upon some of the author's views, with many of which those of our readers with musical bent will be in accord. Who among us has not felt the tedium, not to say boredom, of listening to a three hours' rendering of classical music in a hall which is both overheated and badly ventilated? If music is to become more popular, the public must hear it in purer and cooler air and in reasonably comfortable seats. The author maintains rightly that music should be invigorating and helpful and not morbidly exciting, and many will agree with her when she speaks of some of the ultra-modern harmonic effects as representing "every form of misdirected human energy."

In the second part Dr. Savill deals more directly with the effect of music upon the human system. Here she is a little less convincing because a little less original. The researches of Dogiel, Verdier, Herbert Dixon, and the late Dr. Waller are mentioned as furnishing some scientific basis for the use of music as an auxiliary to the healing art. We should have liked to see some reference to the work undertaken by the Guild of St. Cecilia, now, unhappily, defunct, and also to that of the existing Vocal Therapy Society. There is no doubt whatever, both in the minds of medical practitioners and musicians, that, in capable hands, music is able to play an appreciable part in assisting the recovery from various ailments and in stimulating muscular effort. Excessive motion, stirred up by listening to certain music, may be injurious to the nervous system, but restful harmonies and flowing melodies can be productive of nothing but good. Great music, like great literature, stands the test of time, and in the opinion of the author it is capable of arousing the "same spiritual enthusiasm as can be awakened by prayer, the same exhilaration as love." Dr. Savill has rendered a distinct service to the practitioners of the arts of medicine and of music by showing the practical relationship of one with the other in the pages of this interesting volume.

### THE DOCTOR ON THE BATTLEFIELD.

**Medical Services: General History.** Volume II. By Major-General Sir W. G. MACPHERSON, K.C.M.G., C.B., LL.D. (Stationery Office. £1 1s. net.)

This volume of the History of the Great War is crammed full of information of great value to those whose part it is, or will be, to provide for campaigns to come. In fact, it will be the duty of every medical officer to read the book carefully, Unless its lessons are learnt there can be no doubt that the mistakes made at the beginning of the war will be repeated, and the marvellous evolution that the medical services underwent in the course of hostilities will not be understood and appreciated. This duty cannot be a pleasure, for a more baldly told narrative has seldom been set in type. Nothing enlivens the deadly dulness of the book excepting the photographs. Here and there are hints and suggestions of stories of heroism, of daring deeds, of unselfish sacrifice, yet none is told in such fashion as to stir the blood of the reader or rouse him to emulation. This is a pity, though maybe it is right in an official book of this nature. None the less, if these five hundred pages of unimpeachable facts, perfect tables, painstaking statistics, and carefully drawn plans had been enlivened by some faint touches of literary style, by here and there a little humour, and now and again by a stirring tale, the reading of them would not have been so dreary a task. And if only the compilers had made less use of "but"! There is scarcely a page on which the overworked word does not appear unnecessarily or with a meaning better otherwise expressed.

The volume details the medical arrangements made in France and Belgium from the beginning of the war to the Battle of Loos. The information given is probably as complete as is possible. The work done in digging it out from masses of documents and diaries must have been prodigious and wearying to body and soul. No pains have been spared in any detail, the maps and plans are good and clear, the graphs are easily understandable, the type and printing excellent. It is a book to refer to for facts and has only one fault—it is lifeless in the reading.

### NURSING CANCER.

**How to Nurse Cancer Patients.** By E. P. BARTON, R.R.C. (The Scientific Press. 1s. 3d. net.)

This unpretentious little book has a Preface by Miss Montgomery, Matron of Middlesex Hospital, and therefore herself an authority on the subject of cancer. She points out that having been written by one who has given many years to the service of those who are sick with chronic and incurable diseases, the book will be of the greatest assistance to others who are doing the same. A preliminary word is also added by Sir Bruce Bruce-Porter, who likewise recommends to district nurses the many useful hints contained in the little manual. As Matron in a Poor-Law Infirmary for a long period, its author, Miss Barton, has indeed had unique opportunities of observing the final stages of cancer in the many poor who go there to die, and perhaps this is why she is at her best in the chapters that deal with the nursing treatment of inoperable cases.

Many kindly and clever devices are mentioned that will help to allay the pain and discomfort of this terrible disease. A special chapter is given to the consideration of those in the poor homes visited by nurses in district work. The book also describes the chief diets and treatments that have been tested at the Cancer Hospital, Fulham, and gives all the necessary details of preparation for operation for cancer in various parts of the body and the nursing points to be observed.

### NO BREAKFAST FOR THE FAT.

**Why Be Fat?** By CECIL WEBB-JOHNSON, M.B. (Mills and Boon. 5s. net.)

The medical book for the layman is our only means of competing with the patent medicine vendor and his advertisements. The endless stream of these publications is therefore probably justified. Dr. Webb-Johnson here adds to volumes on kindred topics one upon obesity, and properly insists that the right kind of diet is the only corrective. His analyses of certain patent medicines should remind his more reflective readers that five shillings spent upon a small book may give them better value than five shillings spent upon a small bottle. But the short-cut is the popular desire; and there are no short-cuts to health. It is so much easier to "take something" extra than to go without breakfast, for this is Dr. Webb-Johnson's first prescription.

He believes in electrical treatment for local adiposity if diet is not neglected and sleep not over-prolonged and plenty of walking taken.

We are glad to see the author's praise of riding, which we have heard another medical man (who did ride) call the lazy man's exercise. None, however, exercises every portion of the body so well, and its supreme advantage is that this is accomplished without strain or fatigue. But it is not within the reach of every patient and perhaps not to be learnt in middle life. Golf, on the other hand, may be dangerous, and there is something tragically fitting in the recurrent deaths of fat men upon the golf-courses. As in his other books, the author is explicit and simple, and the suggested dinners that he gives will be of great help to the doctor's most important friend or foe, the patient's family.

### THE MILROY LECTURES.

**Canned Foods in Relation to Health.** By William G. Savage, M.D., D.Ph. (Cambridge University Press. 8s. 6d. net.)

The research work of the County Medical Officer of Health for Somerset is so well known that publication, in this form, of his Milroy Lectures for 1923 will be generally welcomed. We are all much concerned at the present time to know whether the tendency to provide our population with ever-increasing amounts of tinned food is to be encouraged or not. By slight additions to his original lectures Dr. Savage has given us a comprehensive review of the whole subject of canned foods in relation to public health, extending his field of observation considerably beyond our own shores, most notably in Canada and the U.S.A. It is reassuring to learn that he finds canned foods to be undoubtedly safer than fresh materials when these are dealt with in haphazard fashion and under inadequate control. Such dangers as lurk in the canned products can for the most part be readily guarded against, and, provided those foods are not used as main articles of diet, there is little danger of vitamin deficiency.

Danger from actual metallic tin poisoning the author finds to be almost negligible, while, according to his figures, canned foods are not more likely than fresh foods to be vehicles of infection. Their special liability to cause botulism poisoning is serious on account of its fatal nature when it does occur; but actually its incidence is so rare as to be almost insignificant. But Dr. Savage finds that the use of canned foods can only be advocated from the public health point of view if we can be satisfied, not only that the quality is sound, but that the price per 1,000 calories of food value compares favourably with the cost of a similar unit of the fresh food. To divert any considerable proportion of the population's earnings into the buying of canned foods yielding less energy per unit for the same expenditure might have a disastrous effect on the health of the community.

---

The National Clean Milk Society publish a Report of the Proceedings of the National Milk Conference on Pasteurisation, held at the Guildhall on November 21, 1923.

